# Report of U. S. Marine Hospital

**Published:** 1874-05

**Authors:** 


Annual Report of the Supervising Surgeon of the Marine Hospital, Service of the United States, for the fiscal year, 1873. John M. Woodworth, M.D. Washington: Government Printing Office. 1873. Octavo; Cloth, pp. 154.

To the statistical report of the Marine Hospital service for the year, is added, in an appendix, an essay upon Hospitals and Hospital Construction, by Dr. Woodworth, which is well illustrated; an essay upon the Natural History of Yellow Fever in the United States, by Dr. J. M. Toner, of Washington; an essay upon the Yellow Fever Epidemic of 1873, by Dr. Woodworth; Report of a case of Double Diaphragmatic Rupture and Hernia, by Thomas T. Minor, M.D., (illustrated); an essay on Strictures of the Urethra, by C. N. Ellinwood, M.D., and O. L. Crampton, M.D.; an essay on the Sailor and the Service at the port of New York, by Heber Smith, M.D.; and a report upon the River-Boatmen of the Lower Mississippi, by Orsamus Smith, M.D.

